# 

**Published:** 1875-03-01

**Authors:** 


*ORIGINAL COMMUNICATIONS:*

What are Medicines; What the Distinctions between Food and Medicines; Their Classification for Therapeutic Purposes; Ætiology. By N. S. Davis, M.D. ..... 117

*CLINICAL REPORTS:*

Cases Presented to the Clinical Class in the Medical Wards of the Mercy Hospital, Feb. 11-12, 1875. By N. S. Davis, M.D. ..... 126

*TRANSLATIONS:*

Progress of Medical Science in Germany. By E. J. Doering, M.D. ..... 130

*EDITORIAL DEPARTMENT:*

A Medical College in Asiatic Turkey. ..... 132

Rush Medical College. ..... 134

*CORRESPONDENCE:*

General Hospital of Vienna. ..... 135

*SOCIETY REPORTS:*

Chicago Medical Society. Regular Meeting Jan. 4, 1875. ..... 137

*GLEANINGS FROM OUR EXCHANGES:*

Diphtheria and Scarlatina. By D. H. Hayden, M. D. ..... 139

Two Cases of Post-partum Hæmorrhage treated by the Injection of Perchloride of Iron. By W. P. Swain, F. R. C. S. ..... 142

